# Congenital Cleft Palate and Its Treatment

**Published:** 1892-10

**Authors:** P. W. Moriarty

**Affiliations:** Boston


### CONGENITAL CLEFT PALATE AND ITS TREATMENT.[1]

BY P. W. MORIARTY, D.M.D., BOSTON.

Of the primary cause of this distressing deformity we know nothing beyond the fact that there is an arrest of development about the second or third month of fœtal life.

Dr. Kingsley says,[2] "In a large majority of cases they are the solitary instances in the family. It does not seem to any extent to be entailed or transmitted to offspring."

J. Oakley Coles, in "Deformities of the Mouth," says, "Whether cleft palate is hereditary or not it seems impossible to determine,

---

[1] Read at a meeting of the Harvard Odontological Society, held at Boston, Mass., May 26, 1892.

[2] Proceedings of the New York Academy of Medical Sciences, January, 1866.

though from the evidence we have on hand we should come to the conclusion that it is not."

Professor Haughton, of Dublin, thinks diet has some effect on the production of cleft palate, and advised the parents to use food and medicine calculated to promote the formation of bone.

Salter[1] says, "The cause is an arrest of development at an early period of fœtal life, local and very often partial."

Dr. E. S. Talbot,[2] of Chicago, "does not believe in the heredity of cleft palate; if it were so, they should be common among the so-called defective classes." He examined two thousand idiots, eighteen hundred deaf, dumb, and blind, and over eighteen hundred insane, and only found two cases of cleft palate. He thinks "it a purely congenital trouble, the result of defective nutrition. It does not follow that the mother of every child born with cleft palate has not had proper or sufficient food, but the result more frequently comes from perverted function, causing perverted nutrition, and so arrested development."

Dr. Walter Channing, of Brookline, Massachusetts, examined one thousand insane and idiotic patients, and found but three cases of cleft palate.

Dr. T. A. Manley thinks it is due to some nervous influence which we do not understand.

Dr. Edwin Sarcombe[3] says, "Cleft palate must, from the nature of things, have affected the human subject through all time; it must be coequal in antiquity with the other ills to which flesh is heir."

Cases reported which would seem to indicate that it was hereditary are exceptional. When more than one case occurs in the same family, they are simply coincidences; they are the exceptions to the general rule, proving the correctness of the rule that it is not inherited. Except in very rare cases, there is absolutely no evidence of the direct transmission. If it were inherited, there should be a direct and positive evidence of it; it should occur more frequently and the preponderance of evidence should prove it. Scientific men who have seen hundreds of cases, from the knowledge gained from the history of the families in which such cases appeared, agree that it is not inherited.

The theory of heredity is largely a matter of speculation, the

---

[1] "Dental Surgery and Pathology," New York, 1875.

[2] *Dental Cosmos*, vol. xxxii. p. 729.

[3] Transactions of the Medical and Chirurgical Society, London, 1857.

corner-stone of all the aristocracies of the world, an excuse for their failures and vices, concerning which no two of the great writers on the subject agree. All agree that certain diseases can be transmitted, because they are due to a germ, microbe, or morbid poison.

Weismann says that the transmission of acquired characters has no existence in fact, that no botanical facts prove the transmission, and that there are not even any facts which render such transmission probable.

The German philosopher, Kant, and Wilhelm His deny that the transmission of mutilations can ever occur, and agree with Weismann that all the so-called facts in support of such a theory are of no greater value than the merest tales.

Lamarck thought that any change in the structure of any part of an organism was chiefly brought about when the species in question met with new conditions in life and was thus forced to assume new habits.

Darwin added the extremely far-reaching principle of natural selection.

The theory of natural selection does not explain cleft palate. What is there in cleft palate necessary or useful, that nature should desire such a change in the structure of the individual? Those mostly concerned are anxious to remedy it. No theory of any of the writers on heredity will account for the cause of this deformity. It is not a diseased condition due to a morbid poison; it has no tendency to increase in the individual. In a great many patients there is no loss of tissue or bone, simply a displacement. The only logical explanation of the cause is that it is accidental, or due to defective nutrition. The embryo during intra-uterine existence is as liable to accidents as is the individual in after-life.

If cleft palate were transmissible, it should increase in a marked degree; it should reappear in the offspring with sufficient frequency to exclude all possibilities of chance. Changes in the structure of the organism, like cleft palate, do not arise from the germ itself, as the human germ plasm develops into the perfect man. This occurs so often that deformities are rare, are the exception, and must be due to the reaction of the body to accidental or external influences. Nature seeks to avoid instead of transmitting deformities. Nature has a horror of them, and will assert itself and return to the normal development.

Cleft palate is found in children born in the mansions of the rich as well as in the hovels of the poor; in the children of the brainy as well as of ignorant parents. We know it is due to an arrest of development, and science will yet discover what causes the arrest.

We have but few data to base our conclusions on.

I would suggest that blanks based on the following or similar questions be filled out in every case of cleft palate coming into our hands. At some future time we may be able, from observation and the statistics at our command, to throw some light upon the subject.

1. Age.

2. Sex.

3. Assigned cause.

4. Any history of heredity.

5. Mother's condition during gestation.

6. Relationship of parents.

7. Extent of cleft.

8. Condition of teeth.

The child born with cleft palate manages to get nourishment sufficient for existence, and apparently a larger percentage reach adult age than of those with normal palates.

The patients are not conscious of their deformity until they reach the age of six or over; then it dawns upon them, the sneers of their playmates making them aware that it is impossible for them to speak as others do. Then commences a miserable existence for some of the most sensitive of human beings.

The inability to speak correctly is the only and great disadvantage of being afflicted with cleft palate, excepting, of course, a greater tendency to catarrhal troubles.

In 1764 surgery first attempted to remedy this defect, by bringing the parts together. The operation was revived in 1825, since when staphylorraphy was more or less of a success as an operation, but a dismal failure in improving the speech.

The success of mechanical treatment caused surgeons to abandon the operation, but during the last few years the operation has again been revived by German, French, and American surgeons, some of whom claim that the failures to improve the speech was due to a lack of after-training. Another reason given for operating was the expense of a mechanical appliance. This is not a valid reason. No surgeon with the ability to perform an operation will charge a smaller fee than that charged by a dentist for a mechanical appliance, unless he operates upon a charity patient at a hospital; even then, in this city, at least, this is not justifiable, as at the Harvard Dental Hospital we insert appliances for the poor at a nominal fee.

Records show that one in twenty of surgical operations are successful as an operation, but we never see a case where the speech has been made perfect. In fact, the more successful the operation, the greater the injury to the patient, owing to the increased difficulty of mechanical treatment.

The causes of the failure of surgery to improve the speech is that even if the tensor palati muscles are not divided in operating, the palate after an operation is not of sufficient length to close the opening between the cavities of the mouth and nose.

I call your attention to the reports of two cases of only a slight deviation from a normal palate, but in both the power of correct speech was impossible, yet the appearance of both palates was without doubt better than any operation ever could be.

Case I.—Dr. R. Ottolengui[1] reports a case of a palate not cleft, but short, one that would not reach the pharyngeal wall, whose speech could not be understood.

Case II. was a patient of Dr. Hodges, with only a slight fissure of the uvula which rendered the closure of the nasal cavity defective, yet he says, "The palate appeared more perfect than could be obtained by an operation on a congenital case."

Surgeons before operating upon patients should inform them of the success of the dental specialist. Surely in the treatment of cleft palate we all should use common sense, that is, judgment derived from experience, rather than theories, and see facts as they are.

Staphylorraphy should not be performed until surgeons can show or demonstrate that articulate speech can be restored.

Snell, in 1823, was the first who by mechanical means attempted the treatment of congenital cleft palate.

Dr. C. H. Stearn,[2] in 1845, described his appliance for the treatment of cleft palate. Concerning it, Dr. Kingsley says, "His instrument was a marvel of ingenuity. . . . Two principles were vital to Dr. Stearn's instrument,—viz., first, that the artificial velum should embrace the levator muscles of the palate so that it could be lifted by them; and secondly, that it should bridge the upper pharynx behind the uvula and cut off nasal communication at will." The results were marvellous.

Stearn's articles sent a ray of hope to the afflicted, but the appliance was so complicated that his grand idea was lost sight of until Dr. Kingsley, who had given much time and attention to the

---

[1] *Brooklyn Medical Journal*, vol. v. p. 529.

[2] *Lancet*, London, 1845, vol. ii. pp. 7, 260, 284, 310.

subject, met him. The master mind of Kingsley was quick to grasp the correct principles of Stearn, and by his ability and ingenuity was enabled to simplify the appliance.

Dr. Kingsley studied the subject as it never was studied before, and science and unfortunate humanity received the benefit of his great work. The profession of dentistry was ennobled by his labors. Dr. Kingsley as an authority on the subject stands preeminent. He gives Dr. Stearn full credit for being the pioneer in this work.

We who are here to-night are familiar with the Kingsley appliance, owing to the work of our own Dr. Grant, who is as enthusiastic in regard to its merits as Dr. Kingsley himself.

From my own experience, and the experience gained by seeing a large number of cases which Dr. Grant had treated, I am convinced that Dr. Kingsley's appliance is a remarkable success. The only objection to it is that the velum needs renewing every one or two years, the expense of a new velum being five dollars.

Dr. Kingsley advises the wearing of an obturator of hollow vulcanized rubber after the patient has learned to articulate with a soft velum.

Dr. William Suerson,[1] of Berlin, in 1867 called attention to the action of the constrictor muscles of the pharynx in the production of speech, in the use of his appliance. This was not hollow, but was similar in appearance to the Kingsley obturator, or the Baker appliance, if without the hinge-joint. Suerson's appliance was heavy, clumsy, and rigid, depending entirely upon the action of the superior constrictor muscle. Correct speech, unless previously acquired, would be difficult.

In 1880, Dr. H. A. Baker,[2] of Boston, describes an appliance which he was using with great success. The one I now pass around is similar in construction. It consists of the retaining-plate, to which is attached the hollow bulb by means of a hinge-joint.

The original Baker had a delicate spring to keep the bulb in contact with the levator muscles.

The hinge-joint in any hard rubber mechanism is a necessity, as by its means the levator muscles are utilized in the production of speech.

Dr. Parkinson, in the *Lancet*,[3] in 1867, shows a cut of an appli-

---

[1] *American Journal of Dental Science*, December, 1867, and May, 1868.

[2] *Dental Cosmos*, April, 1881. Read before the Massachusetts Dental Society, December 9, 1880.

[3] Vol. i. p. 41.

ance with a hinge and spring, the obturator being made of a flat vulcanite disk. The side view of the same in Salter's Pathology and Surgery[1] very much resembles that of Baker, excepting, of course, that Parkinson's obturator was flat, while Baker's was hollow and of the shape of a chestnut.

Dr. Baker, in his paper read in 1880, describes how he accidentally discovered how to make the bulb hollow, evidently being unaware that Dr. Kingsley described how it was done in the *Dental Cosmos*, May, 1877.

I have seen several of Dr. Baker's patients, and their speech was perfect.

The next appliance which I show you is Dr. Grant's idea, the soft velum of Kingsley being replaced by the underflap and part that goes through the fissure being produced in vulcanite, the upper flap of soft rubber being hooked on to the gold-headed pins screwed into the lower part, this velum-obturator being attached to the retaining-plate by means of a hinge. Dr. Grant uses this occasionally, but prefers the original Kingsley arrangement.

The next one which I pass around is that suggested by Dr. Fillebrown, in which the soft velum of Kingsley is reproduced in hard rubber, and attached to the retaining-plate by means of a strong hinge. This appliance I have used on several patients at the hospital who are at present making rapid progress in acquiring correct speech.

Dr. Fillebrown's ideal obturator, described in the *Independent Practitioner*,[2] we have used at the school in several cases, time will tell with what success. Dr. Fillebrown claims perfect success with it.

The last appliance which I exhibit is the Gibson, described in the INTERNATIONAL DENTAL JOURNAL.[3] He clasps buccal surfaces of second bicuspids and first molars extending between the teeth and vulcanized into the rubber or soldered to a gold plate. The obturator is thin, being only an eighth of an inch thick, made of black rubber. It is rigid, but much lighter than the Suerson. Dr. Gibson claims that he has had great success with it.

I intended to have had a patient here to-night who would wear and speak with each of these appliances, but owing to the short time I have had in preparation, and that the patient was unaccustomed to some of them, I deemed it best to postpone it until a future meeting, when, if you desire it, you can have an opportu-

---

[1] Page 389. [2] Vol. ix. p. 565. [3] July, 1890.

nity of observing the difference, if any, in the speech of the patient while wearing the various forms.

The wonderful success and ingenuity of these men in the treatment of congenital cleft palate reflects honor on the profession as a whole. Those whose labors made this success possible deserve the credit which should be gratefully accorded them. We, their followers, should avoid all false pretences and any approach to quackery. If we invent something new, or improve on an old appliance, in describing it we should not enshroud it in language incomplete in specific details, or seek to keep it a mystery by describing a process which we do not ourselves perform.

To the student who seeks to make himself familiar with the subject I would strongly recommend Dr. N. W. Kingsley's excellent work, "Oral Deformities," and "The Mechanical Treatment of Deformities of the Mouth," by Messrs. Robert Ramsey and James Oakley Coles. These two works will save an immense amount of research.

